# Effects of Parenteral Supplementation with Minerals and Vitamins on Oxidative Stress and Humoral Immune Response of Weaning Calves

**DOI:** 10.3390/ani10081298

**Published:** 2020-07-29

**Authors:** Guillermo Alberto Mattioli, Diana Esther Rosa, Esteban Turic, Sebastián Julio Picco, Santiago José Raggio, Antonio Humberto Hamad Minervino, Luis Emilio Fazzio

**Affiliations:** 1 Laboratorio de Nutrición Mineral, Facultad de Ciencias Veterinarias, Universidad Nacional de La Plata, calle 60 y 118 s/n, 1900 La Plata, Buenos Aires, Argentina; mattioli@fcv.unlp.edu.ar (G.A.M.); drosa@fcv.unlp.edu.ar (D.E.R.); spicco@fcv.unlp.edu.ar (S.J.P.); 2 Biogenésis Bagó, Panamericana Km 38,5, B1619 Garín, Buenos Aires, Argentina; esteban.turic@biogenesisbago.com (E.T.); santiago.raggio@biogenesisbago.com (S.J.R.); 3 Laboratory of Animal Health, LARSANA, Federal University of Western Pará, UFOPA, Rua Vera Paz, s/n, 68040-255 Santarém, Pará, Brazil; 4 LAPEVET-Laboratorio de Patología Especial Veterinaria (FCV-UNLP), Facultad de Ciencias Veterinarias, Universidad Nacional de La Plata, calle 60 y 118 s/n, 1900 La Plata, Argentina

**Keywords:** beef calf, immune response, oxidative stress, weaning, weight gain

## Abstract

**Simple Summary:**

Weaning is a stressful phase for calves; the stress due to separation from the cow results in weight loss and may lead to a decrease in the immune system, making calves more prone to diseases, even in a low-stress (fence-line) weaning system. Mineral and vitamin supplementation can improve the immune system and could therefore be of assistance for calves during weaning. We studied the effects of injectable (subcutaneous) supplementation with copper, zinc, selenium, manganese, and vitamins A and E. Calves were supplemented twice, before weaning and on the day of weaning. We evaluated variables related to the immune status and the immune response to a vaccine containing bovine herpesvirus type 1, as well as body weight and average daily gain. Parenteral supplementation of minerals and vitamins with antioxidant effects in a low-stress weaning system prevented the decrease in variables related to the immune system, improved antibody response, and had positive effects on body weight.

**Abstract:**

We aimed to evaluate the effects of injectable mineral and vitamin supplementation on weaning calves subjected to a low-stress (fence-line) weaning system. Seven-month-old Aberdeen Angus female calves (n = 40, 152 ± 11 kg body weight) from a selenium-deficient area of extensive cattle production on natural grass were randomly assigned to two groups (n = 20 each). One group received subcutaneous supplementation with copper, zinc, selenium, manganese and vitamins A and E (SG), and the other was given sterile saline solution (CG). The animals were supplemented twice, seven days before weaning (day −7) and on the day of weaning (day 0), and they were evaluated 30 (+30) and 60 (+60) days after weaning. Total antioxidant status (TAS), selenium-dependent glutathione peroxidase (GPx) activity, body weight, and average daily gain (ADG) were evaluated. Additionally, antibody titers were assessed prior to and after each immunization with a vaccine containing bovine herpes virus type 1 (BoHV-1). On day +30, body weight (*p* = 0.03) was higher in SG, whereas TAS (*p* = 0.02) and GPx (*p* = 0.0038) activity were lower in CG and remained constant in SG. Antibody titers increased in SG and CG following immunization, being higher in SG on days +30 and +60 (*p* < 0.05). In conclusion, parenteral supplementation of minerals and vitamins with antioxidant effects in a low-stress weaning system prevented the decrease in TAS and GPx activity, improved antibody response and had positive effects on body weight.

## 1. Introduction

Weaning is an important calf management system carried out in beef and dairy farms through different methods [1]. During weaning, calves are exposed to several stressful factors, which can lead to increases in disease susceptibility and reduced production, with decreases in body weight (BW) [2,3]. Weaning stress leads to a catabolic state in which oxygen reactive species are highly produced, posing a challenge for antioxidant defenses in calves [4]. In this context, any alteration in prooxidant/antioxidant balance causes oxidative stress and induces immune dysfunction [5], indirectly affecting growth [6]. Total antioxidant status (TAS) is a parameter used to assess antioxidant defense capacity, including all antioxidant mechanisms [7]. One of the main antioxidant enzymes is glutathione peroxidase (GPx), whose activity depends on selenium (Se), and is therefore used as an indicator of Se status in cattle [8,9]. Selenium deficiency is usually endemic and can affect extensive cattle production without mineral supplementation [10]. This is the case of the Salado River Basin (SRB), the main cattle breeding area in Argentina, producing 2.8 million calves per year and showing low Se concentrations in pasture and cattle plasma [11], together with sporadic cases of nutritional muscular dystrophy [12].

Minerals such as Se, copper (Cu), zinc (Zn), chromium (Cr), and manganese (Mn), as well as vitamins A and E, increase the antioxidant capacity and consequently the immune response in calves [2,13]. Evidence in the literature shows that combined parenteral supplementation with Se, Cu, Zn and Mn improved immunological parameters [14] by increasing antibody response to vaccination against bovine herpes virus type 1 (BoHV-1) in steers [15] and bovine viral diarrhea virus (BVDV) in calves [16]. This type of supplementation also contributed to higher daily weight gain in heifers [17] and steers [18], improved health and antioxidant status and reduced bacterial infections in newborn calves [19]. Likewise, the use of injectable vitamin E and Se in prepartum dairy cattle enhanced antioxidant capacity [20] and decreased the incidence of postpartum mastitis [21]. However, average weight gain (AWG) was not improved in weaning calves [22]. Whereas oral vitamin E supplementation improved the health of calves at feedlot entrance [23], parenteral vitamin E (1 mg/kg) and Se (0.1 mg/kg) supplementation improved immunoglobulin G titers against *Mannheimia haemolytica* in shipping-stressed calves [24]. Injectable vitamin A is not commonly used because cattle fed fresh grass are not normally deficient, even though rare cases of hypovitaminosis A can occur in extensively grazed beef cattle [25,26]. On the other hand, parenteral vitamin A supplementation has been reported to increase weight gain in calves [27]. Similarly, oral vitamin A supplementation after bovine coronavirus (BCoV) immunization increased antibody titers [28].

Previous studies have reported different results regarding the utilization of parenteral supplementation of beef cattle with minerals and vitamins. Swecker et al. [22] evaluated the effects of Se and vitamin E separately or combined, without finding a significant effect on calf growth rate. Arthington et al. [14], using three consecutive intramuscular supplementations of trace minerals (Zn, Mn, Cu, and Se), showed that they increased Cu and Se status without affecting calf BW gain, but increased heifer BW gain and humoral immune response. Conversely, Mattioli et al. [29] found that Cu and Zn supplementation resulted in higher weight gain and higher humoral immune response in pre-weaning calves. Differences in the results obtained could be related to the minerals and vitamins used, the feed condition (deficient, marginal or sufficient mineral/vitamin nutrition), and the supplementation protocols, i.e., different frequencies of supplementation and age of the supplemented animals. Here, we propose an innovative protocol that combines parenteral supplementation of minerals (Cu, Zn, Se and Mn) and vitamins (A and E), which has not been evaluated so far. The protocol was specifically designed for beef calves raised under marginal mineral/vitamin conditions and consists of two consecutive supplementations seven days prior to and on the day of weaning.

In order to minimize weaning stress on calves, a two-step weaning system that promotes better feeding and reduced walking is proposed [30,31,32]. This is the case of fence-line weaning, a method consisting of separating calves from their dams, which are moved to an adjacent paddock [33]. Thus, we aimed to evaluate the effects of parenteral supplementation with minerals (Cu, Zn, Se and Mn) and vitamins (A and E) on animal production, antioxidant status, and humoral immune response in fence-line weaned calves from a farm with reported Se deficiency.

## 2. Materials and Methods

All experimental procedures were approved by the Committee for the Care and Use of Laboratory Animals (CICUAL, for its Spanish acronym), School of Veterinary Sciences, La Plata National University, Argentina (Protocol no. 105-2-20P). The trial was carried out on the experimental farm *Manantiales* (Chascomús, Buenos Aires; 35°44′31.5″ S 58°06′11.7″ W), with characteristics similar to those in the SRB, i.e., poor drainage, floods and higher quantity and quality of grass production during spring.

### 2.1. Animals

Forty healthy Aberdeen Angus suckling female calves were used. Calves were 7 months old, had a BW of 152 ± 11 kg, and were negative to BoHV-1 antibodies. During the study, calves were kept in a paddock with grass of native and naturalized species (*Chaetotropis elonga, Stenotaphrum secundatum, Paspalum dilatatum, Lolium perenne, Lotus tenuis*) and had access to water ad libitum.

### 2.2. Groups and Treatments

A completely randomized design was used. All animals were evaluated at four moments: seven days before weaning (day −7), on weaning day (day 0), and 30 (+30) and 60 (+60) days after weaning. Calves were randomly assigned to two groups with similar age and weight (n = 20 each). On day −7 and day 0, one group (SG) received a mineral supplement containing Cu (10 mg/mL as edetate), Zn (40 mg/mL as edetate), Se (5 mg/mL as sodium selenite) and Mn (10 mg/mL as edetate) (Adaptador Min^®^, Biogénesis Bagó SA, Garín, Argentina), and a vitamin supplement (1 mL/50 kg) containing 3.5% vitamin A retinyl palmitate and 5.0% vitamin E acetate (Adaptador Vit^®^, Biogénesis Bagó SA, Garín, Argentina). The other group (control, CG) received the same volume (1 mL/50 kg) of sterile saline solution on the same days. Weight was recorded early in the morning (7:00 a.m.) without prior fasting at the beginning of the trial (day −7) and on days +30 and +60. The average daily gain (ADG) was calculated on days +30 and +60.

### 2.3. Oxidative Stress Analysis

For oxidative stress evaluation, we analyzed TAS and GPx activity as indicators of Se status. Both variables were analyzed in 10 calves per group due to financial limitations, on days −7 and +60. Measurements were performed in duplicate, and they were repeated when the coefficient of variation was greater than 10%. Blood samples were obtained by jugular venipuncture early in the morning. Blood was collected in tubes containing sodium heparin and kept in a refrigerator (4–5 °C) until they were centrifuged within 6 h after collection. Subsequently, the samples were used to measure whole blood GPx activity and plasma TAS using the Ransel and TAS kits (Randox Laboratories, Crumlin, UK), respectively.

### 2.4. Humoral Immune Response Evaluation

To evaluate the humoral immune response, we assessed the antibody titer to a commercial vaccine. Calves were vaccinated with inactivated BoHV-1 vaccine (Bioqueratogen OLEO MAX^®^, Biogénesis Bagó SA, Garín, Argentina) on days 0 and +30. Blood samples without anticoagulant were stored in a refrigerator for 6 h. Then, they were centrifuged at 1000× *g* within 4 h of collection and stored at −20 °C until analyzed. Antibody titers were measured on days −7, +30 and +60. Serum was used for virus neutralizing (VN) antibody titer calculation, which was performed in primary fetal bovine testis (FBT) cultures using the constant virus-variable serum method. Serial serum dilutions were prepared, and 0.1 mL of diluted samples were mixed with 0.1 mL of virus. Each dilution was tested in four wells containing FBT cell monolayers and adsorbed for 1 h. The unadsorbed virus was removed. Plates were then incubated for 3 days at 37.8 °C and the cytopathogenic effect was read microscopically. Log10 of the reciprocal value of the highest serum dilution in which a cytopathogenic effect was prevented was considered as the VN titer. [34].

### 2.5. Statistical Analysis

Data were analyzed using a mixed model for repeated measures over time with SAS statistical software (9.1). Treatment (SG and CG), time (day) and their interactions were fixed variables and animals were the random variable. The SLICE option of the program was used for mean separation when differences in group (treatment) or time × treatment interaction were significant. Significance for the main effects and their interaction was set at *p* < 0.05. *p*-values < 0.1 were considered as a trend. For statistical analysis, antibody titers were normalized using log transformation.

## 3. Results

All the animals remained clinically healthy during the trial. In CG, GPx activity significantly decreased to half of its baseline concentration on day +60 (Table 1). In SG, mineral and vitamin supplementation maintained the same TAS level after weaning (day +60) compared with baseline (day −7). By contrast, CG had a marked decrease in TAS on day +60, reducing to one third of its baseline value. Both TAS and GPx activity were higher in SG on day +60 when compared with CG (*p* < 0.05).

Body weight showed time × treatment interaction (*p* = 0.03; Table 1). Differences were established on day +30 (trend; *p* = 0.09), but not on day +60 (*p* = 0.13). When considering ADG, a time × treatment interaction trend was observed (*p* = 0.07), with a higher ADG in SG only on day +30 (*p* = 0.024).

**Figure 1:**
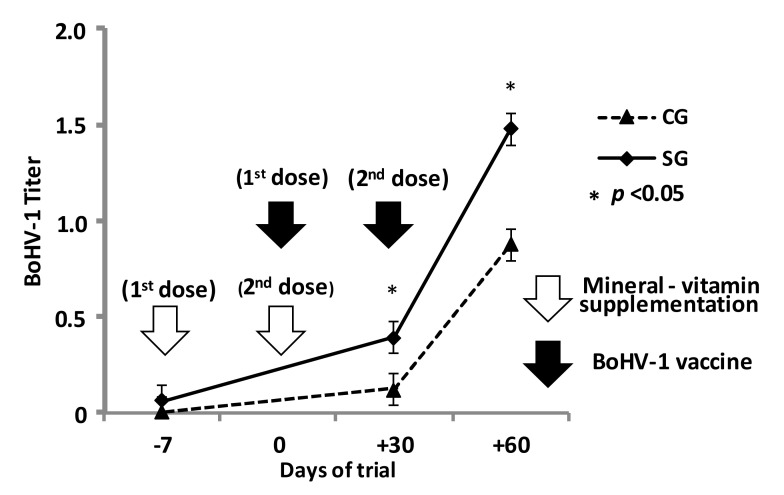
Log-transformed titer responses to BoHV-1vaccine in weaning calves (n = 10 per group). SG, supplemented group (1 mL/50 kg containing 10 mg% Cu, 40 mg% Zn, 0.5 mg% Se, 10 mg% Mn, 3.5% retinol palmitate and 5.0 % vitamin E acetate on days −7 and 0); CG, control group (saline sterile solution on days −7 and 0). SG and CG were vaccinated with inactivated BoHV-1 on days 0 and +30.

The response to the inactivated BoHV-1 vaccine also changed with supplementation. Antibody titers increased with the second dose in both groups, but they were higher in SG at both evaluated moments (day +30 and day +60) (Figure 1).

## 4. Discussion

It is known that any situation causing stress produces oxygen reactive species that inhibit antioxidant enzymes and can produce oxidative damage [4]. Thus, the presence of several stressors worsens the situation and is likely to increase their impact [30]. In high-stress weaning systems, including transport and feedlot entrance, antioxidant capacity is reduced, and it is only restored 28-60 days post-weaning [35,36]. Even though stressors were minimized in the present study, weaning decreased TAS to a third in non-supplemented animals, which is in agreement with previous findings indicating that cow-calf separation itself induced higher social stress [37].

Whole blood GPx activity was constant in SG, despite the two Se doses administered; however, it decreased in CG. These results differ from the increased whole blood GPx activity reported in other stress models, such as calving and early lactation in cows [38,39]. According to these authors, high oxidative stress stimulates GPx synthesis when Se requirements are met by the diet and even when prepartum Se supplementation is higher than required, increasing postpartum GPx activity even more [39]. However, when the diet does not provide enough Se, which is typical of extensive production systems without supplementation, GPx levels remain the same or even decrease [10]. The marginal GPx activity observed at the beginning of the current trial warned us about the risk of Se deficiency [40]. Thus, 60 days after Se supplementation was an adequate time to evaluate differences in GPx activity between SG and CG, as reported in other studies performing such assessment 45 and 75 days after Se supplementation [41,42]. Whereas whole blood GPx activity is a long-term indicator of Se status, plasma and white blood cell Se levels reflect short-term Se status [43,44], probably because Se is incorporated into the erythrocyte during erythropoiesis, as GPx activity is conditioned by its long half-life [9,42]. It is noteworthy that diets supplemented with organic and inorganic forms of selenium produced different alterations on oxidative stress and Se status [45,46]. Further studies are required to evaluate the differences related to parenteral and dietetic Se supplementation.

In this trial, body weight and AWG changes were considered as health indicators, since the number of animals used, besides being appropriate for the immune and antioxidant response, was limited to evaluate productive responses in cattle due to the great variances observed, especially during weaning [2]. When protein oxidation occurs, the body might use energy to generate antioxidant defenses rather than to develop tissue [47]. Furthermore, it has been demonstrated that protein oxidation is associated with reduced feed efficiency [48]. In this sense, injectable trace minerals improved feed efficiency in feedlot calves, with [13] or without [49] an increase in ADG. Likewise, mineral (Cu and Zn) supplementation produced a marked increase in ADG of buffalos from a deficient area [50]. Combined stressing factors led to deeper adaptation changes, with concomitant productive and immunological consequences [30]. In weaning heifers, injectable trace mineral supplementation increased ADG when weaning stress was minimized; however, this effect was not observed when heifers were weaned and then submitted to transport stress [14]. In steer beef calves transported over 3106 km, oxidative damage assessed on plasma malondialdehyde levels was three times higher [35]. It could therefore be assumed that injectable trace mineral supplementation might be beneficial for weaning calves, even though its efficacy would depend on the stress generated. In this context, combined vitamin injection could have a role, considering that vitamin E was effective in reducing the consequences of feedlot entrance [51], although its effect disappeared when its concentration exceeded the requirements [23].

Titers of BoHV-1 have been used as immune capacity markers during stress periods [29,34]. In this trial, the differences in BoHV-1 titers found in SG confirmed the importance of mineral and vitamin supplementation with antioxidant effects before and during weaning stress. Similar studies using modified live virus vaccines with higher antigenicity have shown higher [15] or unchanged [16] BoHV-1 titers with increased BVDV 1 and 2. Although these vaccines are banned in Argentina and only inactivated virus (with lower antigenic capacity) can be used [52,53], BoHV-1 titers generated by the inactivated vaccine were an accurate marker of humoral immune capacity in this trial, as well as in a previous one evaluating Cu deficiency in calves [29].

## 5. Conclusions

Parenteral supplementation combining minerals and vitamins preserved TAS and GPx activity levels in fence-line weaned female calves. The implemented protocol with two consecutive mineral and vitamin supplementations seven days before and on the day of weaning increased humoral immune response and had a positive effect on calf body weight. The protocol proposed here can be recommended for beef cattle management systems raised under marginal conditions of mineral and vitamin intake.

## Figures and Tables

**Table 1:** Total antioxidant status, glutathione peroxidase activity, body weight and average weight gain in weaned calves supplemented with minerals and vitamins or sterile saline solution.

Day	SG	CG	SEM	*p*-Value		
	LSM			Treatment	Time	Treatment × Time
TAS (µg/dL)						
−7	1.07	0.76				
+30	ND	ND	0.17	0.02	0.15	0.25
+60	1.00 ^a^	0.27 ^b^				
GPx (U/g Hb)						
−7	113.8	85.8				
+30	ND	ND	11.55	0.004	0.15	0.10
+60	117.0 ^a^	45.6 ^b^				
Body weight (kg)						
−7	153	152				
+30	165 ^a^	160 ^b^	2.21	0.23	0.0001	0.04
+60	168	163				
ADG (kg/day)						
−7	ND	ND				
+30	0.328 ^a^	0.197 ^b^	0.039	0.15	0.0001	0.07
+60	0.855	0.101				

SG, supplemented group (1 mL/50 kg containing 10 mg% Cu, 40 mg% Zn, 0.5 mg% Se, 10 mg% Mn, 3.5% retinol palmitate and 5.0 % vitamin E acetate on days −7 and 0); *CG*, control group (saline sterile solution on days −7 and 0); LSM, least square mean; SEM, standard error of the mean; TAS, total antioxidant status; GPx, peroxidase glutathione; AWG, average weight gain; ND, not determined. ^a,b^ Different superscript letters in the same row indicate *p* < 0.05 SLICE option.
